# Double carbapenemases in *Klebsiella pneumoniae* blood isolates: dissemination in a single medical center via multiple plasmids and a variety of highly efficient clones

**DOI:** 10.1128/aac.01462-24

**Published:** 2025-02-03

**Authors:** Anastasia Rigatou, Ayorinde O. Afolayan, Elizabeth-Barbara Tatsi, Ioannis Deliolanis, Athanasios Michos, Sandra Reuter, George L. Daikos

**Affiliations:** 1 Department of Microbiology, Laiko General Hospital, Athens, Greece; 2 Institute for Infection Prevention and Control, Medical Center - University of Freiburg 14879 , Freiburg, Germany; 3 Infectious Diseases Research Laboratory, First Department of Pediatrics, “Aghia Sophia” Children’s Hospital, National and Kapodistrian University of Athens 68993 , Athens, Greece; 4 First Department of Medicine, Laiko General Hospital, National and Kapodistrian University of Athens 58836 , Athens, Greece; University of Fribourg, Fribourg, Switzerland

**Keywords:** double carbapenemases, *Klebsiella*, sequence types, plasmids, AMR genes

## Abstract

Acquisition of multiple carbapenemase genes by *Klebsiella pneumoniae* (Kp) is an emerging public health threat. Here, we aim to elucidate the population structure of Kp blood isolates carrying two different carbapenemase genes and identify the mechanism facilitating their dissemination. The study was conducted in a tertiary healthcare center between 2014 and 2022. Twenty-four patients with bacteremia caused by Kp carrying two different carbapenemase genes were identified. All 24 blood isolates were analyzed by short-read genome sequences supplemented by long reads in a selected number of isolates. All isolates carried *bla*_KPC_ (23 *bla*_KPC-2_, 1 *bla*_KPC-3_) and *bla*_VIM-1_ genes, along with a variety of antimicrobial resistance determinants. The isolates were clustered in six clonal lineages (ST39, ST147, ST323, ST258, ST3035, and ST340). Long-read genome sequences demonstrated that each carbapenemase gene was located in a separate group of plasmids: the *bla*_KPC-2_ on a fusion of IncFIB(pQil) and IncFII(K) plasmids, the *bla*_KPC-3_ on IncX3, the *bla*_VIM-1_ on IncC, or a fusion of the IncFIB(pNDM-Mar) and IncHI1B(pNDM-MAR) plasmids. Comparison of plasmid content of eight isolates carrying a single carbapenemase gene from a previous study with eight isolates carrying two carbapenemase genes from the present study, matched by clonal lineages, revealed that the second carbapenemase gene was acquired by addition of another plasmid. Identical plasmids were found within the same lineage and across lineages. These findings suggest that dissemination of carbapenemase genes in our hospital setting was driven by multiple plasmids across a variety of highly efficient clones.

## INTRODUCTION

Carbapenem-resistant *Klebsiella pneumoniae* (CR-Kp) has been established as an important nosocomial pathogen worldwide causing serious infections associated with increased morbidity and mortality (1–3). Resistance to carbapenems is mainly mediated by carbapenemases, enzymes that hydrolyze carbapenems and most clinically available β-lactams, encoded on self-transmissible plasmids and a variety of mobile genetic elements (MGEs) (4, 5). The most commonly encountered carbapenemases are the Ambler class A and D serine-β-lactamases, KPC- and OXA-48-like, respectively, as well as the class B zinc-dependent metallo-β-lactamases (MBLs), VIM, IMP, and NDM, the frequency of which varies by geographic region (6) .The extensive dissemination of CR-Kp is due to the global expansion of highly successful clones, especially clonal group (CG) 258 (i.e., ST258, ST11, and ST512) and CG14. The epidemiology, however, has been changing. New clones are emerging (i.e., ST307 and ST147) and being added to the family of successful clones, indicating an ongoing dynamic process (7).

In Greece, a country that has been affected the most by CR-Kp (8), three high-risk lineages were predominantly circulating within healthcare facilities—ST147, ST258, and ST11—which carried mainly the *bla*_VIM_, *bla*_KPC_, and *bla*_NDM_ carbapenemase genes, respectively (9, 10). A more recent surveillance study, however, has shown the emergence and expansion of new clones (i.e., ST39 and ST323) and a rise in the proportion of those producing metallo-β-lactamases (11). More importantly, 8% of carbapenem non-susceptible isolates co-carried two carbapenemase genes, making these pathogens nearly untreatable. Double carbapenemase production in *Klebsiella pneumoniae* (Kp) was detected for the first time in Greece in 2009 (12). Since then, their presence has been sporadic (13–17). After 2018, however, comparable with the global trend (6, 18), an increasing proportion of CR-Kp co-carry two carbapenemase genes in our geographic region (11, 19). In the present study, we aim to obtain more detailed information on the population structure, the resistome, the virulence factors, and the plasmid content of 24 blood isolates carrying two carbapenemase genes derived from a tertiary healthcare center located in Athens, Greece.

## RESULTS

### Patient population

A total of 26 patients with bacteremia caused by Kp-producing double carbapenemases were identified from our laboratory surveillance records. Of those 26 patients, 24 were confirmed by whole-genome sequencing (WGS) to be infected with isolates carrying two carbapenemase genes: 1 patient was admitted to the hospital in 2014, 1 in 2018, 3 in 2020, 11 in 2021, and 8 in 2022. All episodes of bacteremia were either hospital acquired (*n* = 23) or healthcare associated (*n* = 1). The median duration of hospitalization before the onset of bacteremia was 22 days (mean: 30 days, range: 1–112 days). As shown in Table 1, 13 patients were males and 11 were females with a mean age of 71.3 years (median: 73 years, range: 45–95 years). All patients had more than one underlying disease with a median Charlson comorbididy index of 4 (range: 2–9), and four patients had neutropenia (<1,000 neutrophils/μL) at the onset of bacteremia. More than half (13 of 24) of the infections occurred in intensive care unit (ICU) patients; 5 occurred in patients admitted to medical wards, 3 in surgical wards, and 3 in the hematology unit. The source of bacteremia was the lung in eight patients, the intra-vascular catheter in five, the urinary tract in four, and the abdomen in two. In five patients, no definitive entry could be identified. Eleven patients presented with septic shock at the onset of bacteremia. Antibiotic therapy was selected at the discretion of the attending physician. For empirical therapy, only two patients received treatment with at least one *in vitro* active drug within 48 h after the onset of bacteremia, while the remaining 22 patients received inappropriate empirical treatment. For definitive treatment, 16 patients received treatment with at least one *in vitro* active drug, while 5 patients received inappropriate treatment; 3 of the latter patients were infected with extensively drug-resistant pathogens for which there was no available active drug. Three patients died within 48 h before the susceptibility results were available (Table 1). Overall, the all-cause mortality of 28 days was 62.5% (15 of 24 patients died), 50% (8 of 16 patients died) for those who received at least one active drug, and 80% (4 of 5 patients died) for those who received no active drug.

**TABLE 1:** Clinical characteristics of 24 patients with bacteremia caused by *Klebsiella pneumoniae* carrying two carbapenemase genes

Characteristic	No. (%)
Age (yr), median (range)	73.0 (45–95)
Sex	
Male	13 (54.0)
Female	11 (46.0)
Ward at onset of bacteremia	
ICU	13 (54.0)
Non-ICU	11 (46.0)
Charlson comorbididy index, median (range)	4 (2–9)
Neutropenia at onset of bacteremia	
Yes	4 (16.7)
No	20 (83.3)
Septic shock at onset of bacteremia	
Yes	11 (46.0)
No	13 (54.0)
Source of bacteremia	
Secondary	14 (58.3)
Respiratory	8
Urinary	4
Intra-abdominal/biliary	2
Intra-vascular catheter	5 (20.85)
Primary (no source identified)	5 (20.85)
Empirical treatment	
No active drug	22 (91.7)
At least one active drug	2 (8.3)
Definitive treatment^*a*^	
No active drug	5 (23.8)
At least one active drug	16 (76.2)
Ceftazidime/avibactam + aztreonam	7
Colistin	3
Colistin + tigecycline	2
Colistin + fosfomycin	1
Amikacin	2
Tigecycline + fosfomycin	1

^*a*^ Three patients died within 48 h from the onset of bacteremia before the susceptibility results were available.

### Antimicrobial susceptibility profile

All 24 isolates were resistant to ciprofloxacin, co-trimoxazole, extended-spectrum cephalosporins, carbapenems, and ceftazidime/avibactam. The majority of isolates were also resistant to gentamicin 87.5% (21 of 24), amikacin 70.8% (17 of 24), colistin 54.2% (13 of 24), and fosfomycin 91.7% (22 of 24), and 18 of the 24 (75.0%) isolates had tigecycline MIC of >1 mg/L. The most active agents were cefiderocol and aztreonam/avibactam, although a few isolates exhibited resistance to these newer antimicrobials: 21% (5 of 24) and 4% (1 of 24), respectively (Table 2).

**TABLE 2:** Genomic and phenotypic characteristics of 24 *Klebsiella pneumoniae* blood isolates co-carrying two carbapenemase genes^*a*^

	ST39 (*n* = 9)	ST147 (*n* = 6)	ST323 (*n* = 4)	ST258 (*n* = 2)	ST3035 (*n* = 2)	ST340 (*n* = 1)
K type-K locus (*n*)	K23-KL23 (*wzi*83) (9)	K64-KL64(*wzi*64) (6)	K21-KL21(*wzi*262) (4)	K106-KL106 (*wzi*29) (1), K107-KL107 (*wzi*154) (1)	K25-KL25 (*wzi*133) (2)	K15-KL15 (*wzi*50) (1)
O type-O locus (*n*)	O1-O1/O2v2 (9)	O2a-O1/O2v1 (6)	O3b-O3b (4)	O2afg – O1/O2v2 (2)	O1-O1/O2v1 (1), O2a-O1/O2v1 (1)	O4-O4 (1)
Yersiniabactin (*n*)	*ybt*14, ICE*Kp5* (9), *ybST*:151 (9)	*ybt* 10, ICE*Kp4* (5), *ybST*: 384 (5)	ybt 14, ICE*Kp5* (4), *ybST*:144–4LV (4)	*ybt* 13, ICE*Kp2* (2), *ybST*: 299 (2)	ND	ND
Plasmid types (*n*)	IncFII (9), IncFIC(FII) (9), IncA/C2 (9), ColRNAI (9), IncFIB (9)	IncHI1B (6), IncFII (6), IncFIB (6), ColRNAI (6)	IncFII (4), IncFIB (4), IncA/C2 (4), IncR (4), ColRNAI (4), IncN (4), IncHI1B (4)	IncFII (1), IncFIB (1), IncX3 (2), IncA/C2 (2), ColRNAI (1)	IncFII (2), IncFIB (2), IncA/C2 (2), IncR (1), ColRNAI (2)	IncHI1B (1), IncFIB (1), IncFII (1) IncFIC (FII) (1), Col(IRGK) (1)
Genetic determinants conferring resistance to different groups of antibiotics and resistance phenotypes to respective antibiotics						
β-Lactams	*bla*_KPC-2_ (9), *bla*_VIM-1_ (9), *bla*_SHV-11_ (9), *bla*_TEM-1D_ (6), *bla*_TEM-104_ (1)	*bla*_KPC-2_ (6), *bla*_VIM-1_ (6), *bla*_SHV-11_ (6), *bla*_CMY-13_(6), *bla*_OXA-1_ (5), *bla*_TEM-1D_ (2)	*bla*_KPC-2_ (4), *bla*_VIM-1_ (4), *bla*_SHV-1_ (4), *bla*_TEM-1D_ (4), *bla*_VEB-1_ (3), *bla*_OXA-10_ (3)	*bla*_KPC-2_ (1), *bla*_KPC-3_ (1), *bla*_VIM-1_ (2), *bla*_SHV-11_ (2), *bla*_VEB-1_ (1), *bla*_OXA-10_ (1)	*bla*_KPC-2_ (2), *bla*_VIM-1_ (2), *bla*_TEM-1D_ (2), *bla*_SHV-11_ (2)	*bla*_KPC-2_ (1), *bla*_VIM-1_ (1), *bla*_SHV-11_ (1), *bla*_CMY-13_ (1)
Cefiderocol R (*n*)	(0)	(0)	(4)	(1)	(0)	(0)
AZT-AVI R (*n*)	(0)	(0)	(1)	(0)	(0)	(0)
Polymyxins	*mgrB*-51% (9)	ND	ND	*mgrB*-60% (1)	*mgrB*-0% (1)	ND
Colistin R (*n*)	(3)	(3)	(3)	(2)	(1)	(1)
Aminoglycosides	*aac(6′)-Ib* (9), *aac(6′)-II* (9), *aph3-la* (8), *aac(3)-IId* (6), *aadA2* (3), *strA* (6), *strB* (9)	*aac(6′)-Ib-cr* (6), *aac(6′)-II* (6), *aadA2* (6), *ant(2″)-Ia* (6), *aadA1* (5), *aph3-la* (4), *aadA* (1)	*aac(6′)-II* (4), *ant(2″)-Ia* (4), *aph3-la* (2), *rmtB* (4), *strA* (3), *strB* (4)	*aac(6′)-Ib* (2), *aac(6′)-II* (2), *aadA* (1), *aadA2* (2), *ant(2″)-Ia* (1), *aph3-la* (2), *strA* (2), *strB* (2), *rmtB* (1)	*aac(6′)-II* (2), *aac(6′)-Im* (2), *aph(2″)-IIa* (2)	*aac(6′)-II* (1), *aadA1* (1), *aadA2* (1), *ant(2″)-Ia* (1), *aph3-la* (1)
Amikacin R (*n)*	(9)	(0)	(4)	(2)	(1)	(1)
Gentamicin R (*n*)	(6)	(6)	(4)	(2)	(2)	(1)
Fluoroquinolones	*gyrA-83I* (9), *gyrA-87N* (9), *parC-80I* (9), *qnrS1* (7)	*gyrA-83I* (6), *parC-80I* (6), *qnrA1* (3)	*gyrA-83F* (4), *qnrS1* (1)	*gyrA-83I* (2), ParC-80I (2)	*qnrS1* (2)	*gyrA-83I* (1), *parC-80I* (1), *qnrA1* (1)
Ciprofloxacin R (*n*)	(9)	(6)	(4)	(2)	(2)	(1)
Phenicols	*cmlA1* (3)	*catB4* (6)	*cmlA5* (4)	*catA1* (2), *cmlA5* (1)	ND	ND
Not tested						
Tetracyclines	t*et(A)* (9), *tet(M)* (3)	*tet(A)* (6)	*tet(A)* (4), *tet(G)* (4)	*tet(A)* (2), *tet(G)* (1)	ND	ND
Tigecycline R (*n*)	(8)	(6)	(4)	(2)	(0)	(1)
Sulfonamides	*sul1* (9), s*ul2* (9), *sul3* (9)	*sul1* (6; double copies *n* = 2)	*sul2* (4)	*sul1* (1), *sul2* (2)	*sul1* (2), *sul2* (2)	*sul1* (1; double copies)
Trimethoprim	*dfrA1* (9), *dfrA12* (3)	*dfrA1* (6)	*dfrA1* (4), *dfrA23* (4)	*dfrA1* (2), *dfrA12* (2)	*dfrA1* (2)	*dfrA1* (1)
TMP-SMX R (*n*)	(9)	(6)	(4)	(2)	(2)	(1)
Macrolides	*mphB* (9)	*mphA* (3)	*mphB* (4)	*mphA* (2), *mphB* (1)	*mphB* (2)	*mphA* (1)
Not tested						
Rifamycins	ND	ND	*arr-2* (4)	*arr-2* (1)	ND	ND
Not tested						
*ompK*	*ompK36GD* (9), *ompK35*-7% (1)	ND	ND	*ompK35*-25% (2), *ompK36GD* (1), *ompK36TD* (1)	*ompK35*-23% (2), *ompK36*-7% (1)	ND

^*a*^ AZT-AVI, aztreonam-avibactam; TMP-SMX, trimethoprim-sulfamethoxazole; R, resistance; ND, none detected; numbers in parentheses indicate the number of isolates.

### Antibiotic resistance determinants

All 24 isolates carried *bla*_KPC_ (23 *bl a*_KPC-2_ and 1 *bla*_KPC-3_) and *bla*_VIM-1_ genes. Apart from the two carbapenemases, all isolates co-carried a variety of antimicrobial resistance (AMR) determinants with a mean resistance score of 2.5, resulting in extensively drug-resistant phenotypes (Table 2). All isolates harbored at least one beta lactamase gene (e.g., *bla*_SHV-11_, *bla*_VEB-1_, *bla*_OXA-10_, and *bla*_CMY-13_), in addition to *bla*_KPC_ and *bla*_VIM_, more than one mechanism conferring resistance to fluoroquinolones (*gyrA* and/or *parC* mutations and/or plasmid-mediated *qnr* genes) and several aminoglycoside-modifying enzyme genes [*aac(6′)-Ι*, *aac(6′)-ΙΙ*, *aph(3)-Ia*, and *aac(3)-2d*]. Of note, all four ST323 isolates carried the 16S-rRNA methylase *rmtB* gene that is able to confer resistance to all aminoglycosides with the exception of streptomycin. The bifunctional enzyme gene *aac(6')-Ib-cr*, a variant of *aac(6')-Ib*, can also inactivate fluoroquinolones through acetylation and was found in six ST147 isolates. Also, truncations in genes encoding for outer membrane proteins, OmpK_35_ and OmpK_36_, were noted in 13 isolates (ST39 = 9, ST258 = 2, and ST3035 = 2). Truncations of varying lengths in the *mgrB* gene, which is associated with resistance to colistin (20), were identified in 11 isolates. However, no concordance was observed between phenotypic and genotypic resistance, as out of 13 isolates with phenotypic resistance, only 5 carried a truncated *mgrB* gene. On screening for other colistin resistance genes (e.g., *mcr*) or mutations in genes previously associated with resistance to colistin (e.g., *phoQ*, *crrA*, and *crrB*) (21), none of these was observed (22, 23).

### Sequence types

The 24 isolates with double carbapenemase genes were clustered in six clonal lineages: ST39 (*n* = 9), ST147 (*n* = 6), ST323 (*n* = 4), ST258 (*n* = 2), ST3035 (*n* = 2), and ST340 (*n* = 1).

### Capsular (K), lipopolysaccharide (O) serotypes and virulence determinants

As for the capsular biosynthesis locus (KL) and the lipopolysaccharide O types, there was strong association with the sequence types (STs). In particular, ST39 was associated with KL23, O1; ST147 with KL64, O2a; ST323 with KL21, O3b; ST258 with KL106 or KL107, O2afg; ST3035 with KL25, O1 or O2a; and ST340 with KL15, O4. Concerning the genetic traits associated with virulence, we detected only the yersiniabactin-encoding locus (*ybt*) in 20 of the 24 (83.3%) isolates.

### Plasmid analysis

**Fig 1:**
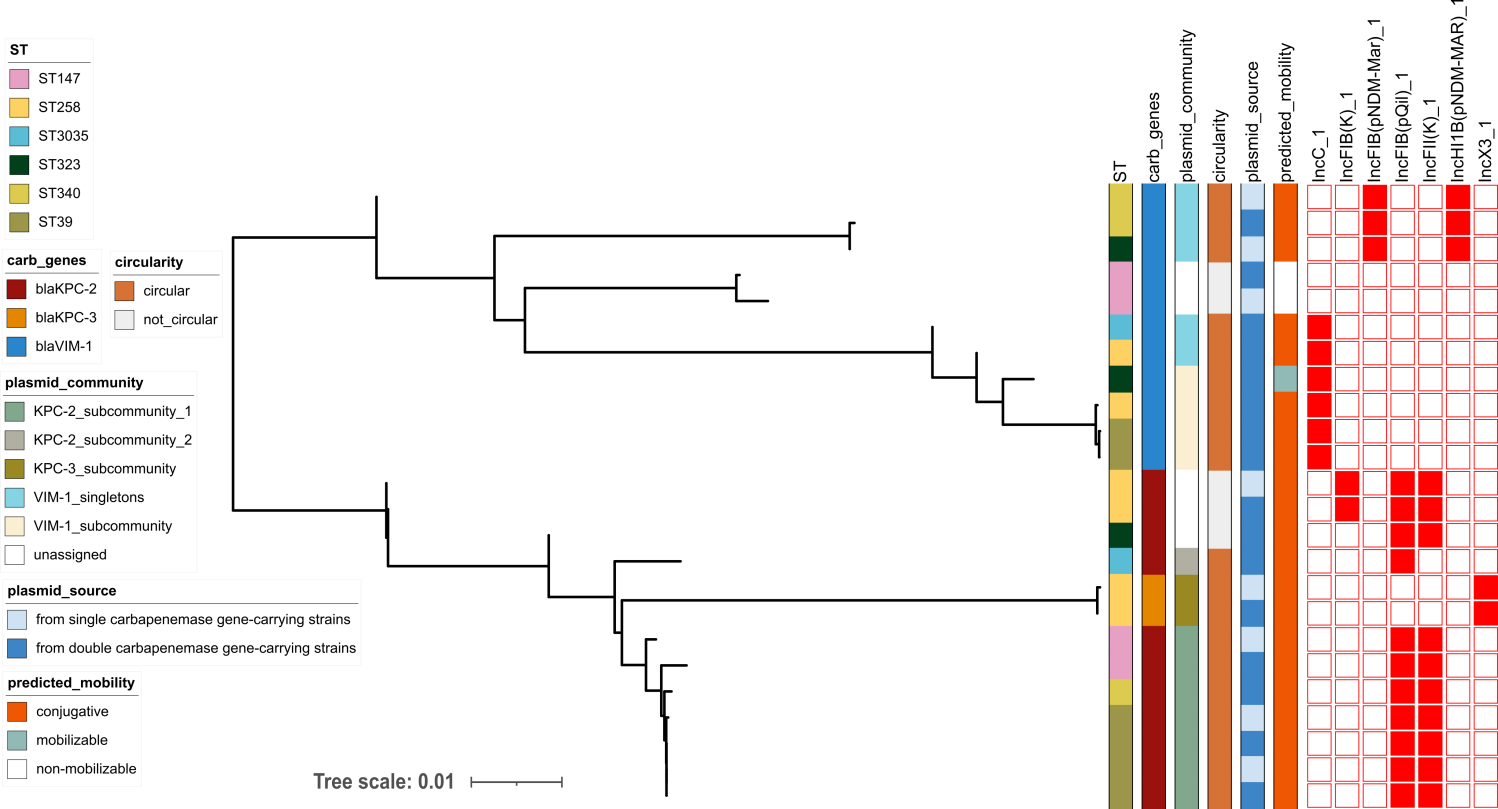
Midpoint-rooted phylogeny of carbapenemase gene-carrying plasmids based on mash distances, accompanied by relevant metadata. Plasmid community networks were not computed for non-circular plasmids. The plasmids were detected in eight *Klebsiella pneumoniae* (Kp) isolates carrying a single carbapenemase gene from a previous study (10) and in eight Kp isolates carrying double carbapenemase genes, matched by sequence type (ST), from the present study and analyzed by long-read sequencing. Different STs, carbapenemase genes, plasmid community, circularity, and source are represented in different colors and intensities. Red denotes the presence of the Inc plasmid type.

Among the isolates carrying two carbapenemase genes, the *bla*_KPC-2_, *bla*_KPC-3_, and *bla*_VIM-1_ genes were each located on separate plasmids (Fig. 1). Of the 24 carbapenemase gene-carrying plasmids, all but 3 *bla*_VIM-1_ plasmids were presumably conjugative (Fig. 1; Table S2). By comparing the plasmid content of isolates of the same lineage carrying one and two carbapenemase genes, our analysis revealed that the acquisition of a second carbapenemase gene was mediated by successive possession of a new plasmid (Table S2). Looking at the number of resistance genes per plasmid (Fig. S1), we found that the acquisition of a second carbapenemase plasmid may co-introduce a number of other resistance genes, depending on the plasmid. *Bla*_VIM_-carrying plasmids were larger in size and contained several antimicrobial resistance genes compared to *bla*_KPC_ plasmids, which predominantly carried only the *bla*_KPC_ gene.

Using Pling (24), circularized carbapenemase gene-carrying plasmids were classified into four communities. One community comprised *bla*_KPC-2_-carrying plasmids (Fig. S2A), another consisted of *bla*_KPC-3_-carrying plasmids (Fig. S2D), and two communities included *bla*_VIM-1_-carrying plasmids (Fig. S2B and C). These communities were further divided into nine sub-communities based on containment distance (shared content as a fraction of the smaller plasmid, with a threshold of 0.3 for plasmid transmission) and Double-Cut-Join-Indel distances between the plasmids (a measure of plasmid relatedness based on structural evolution, with a threshold of 4) (24). The *bla*_KPC-2_-carrying plasmid community was divided into two sub-communities; the *bla*_VIM-1_-carrying plasmids were split into six sub-communities, while the *bla*_KPC-3_ plasmid community consisted of one community.

**Fig 2:**
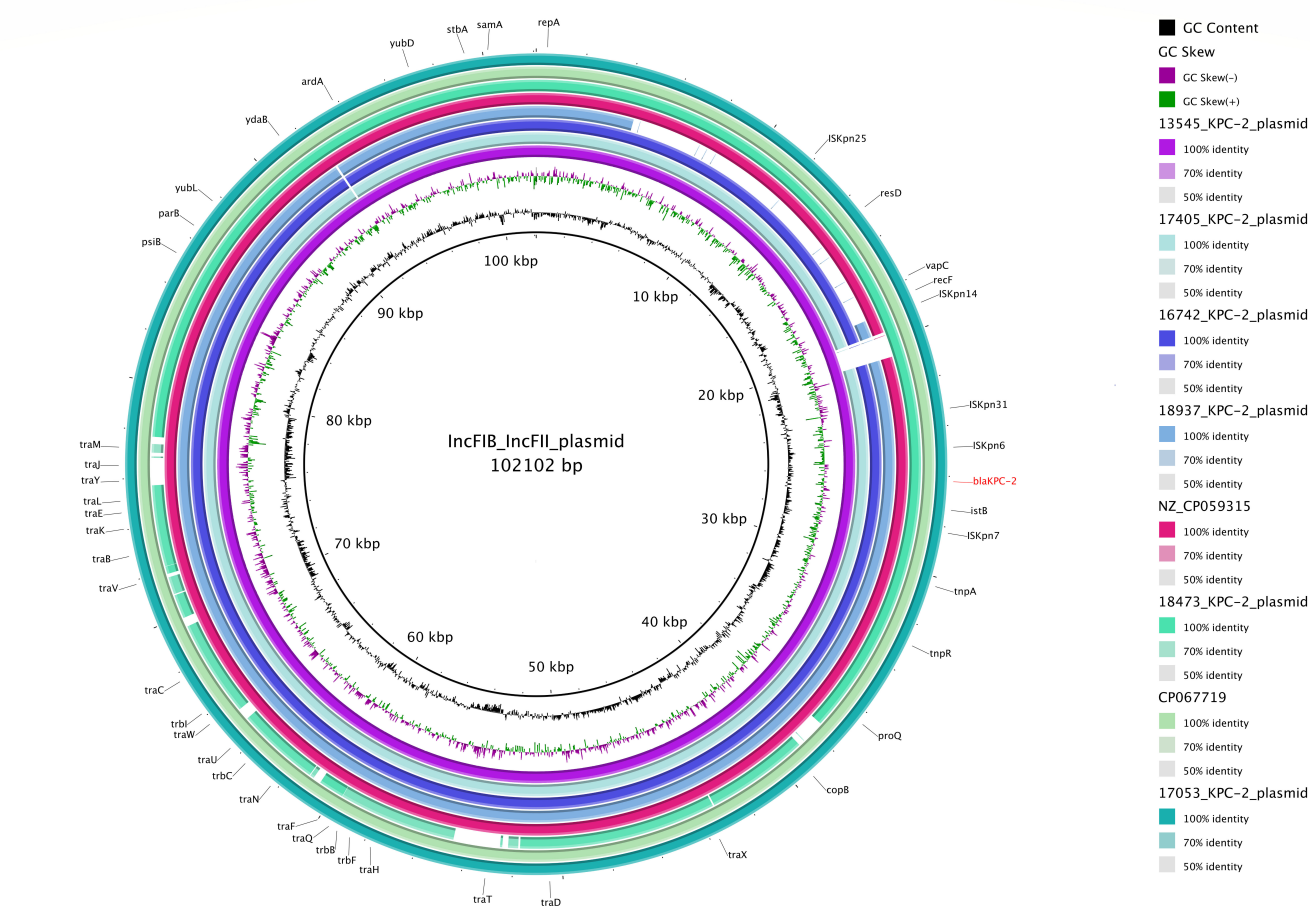
Comparative genomic analysis of *bla*_KPC-2_-carrying plasmids. The *bla*_KPC-2_ gene is indicated in red. Flanking the *bla*_KPC-2_ gene are genes defining the Tn*4401* mobile transposon (composed of *bla*_KPC-2_ gene, transposase gene *tnpA*, resolvase gene *tnpR*, and two insertion sequences, IS*Kpn6* and IS*Kpn7*).

The larger *bla*_KPC-2_ plasmid sub-community comprises plasmids with identical genetic backbones (Fig. 2), with plasmid size ranging from 86 to 102 kbp and estimated copy number 0.86–2.18 per cell (Table S2). These plasmids, characterized by a fusion of IncFIB(pQil) and IncFII(K) plasmid types, are shared among Kp strains belonging to sequence types 39, 147, and 340 (Fig. 1 and Microreact link). Apart from the *bla*_KPC-2_ plasmid with id 17053 which carried an additional *bla*_TEM-1_ gene, all plasmids within this sub-community carried *bla*_KPC-2_ on a Tn*4401a* transposon (variant one or unknown) as the sole antimicrobial resistance gene (Fig. S3). These findings support the transmission of this plasmid among multiple single- and double-carbapenemase gene-carrying Kp lineages.

The *bla*_KPC-3_-carrying plasmid community includes plasmids derived from ST258 strains carrying either a single carbapenemase gene (17,266; plasmid size: 53 kbp) or double carbapenemase genes (18,471; plasmid size: 55 kbp) (Fig. S4; Fig. 1; Plasmid Microreact link). The 18,471 *bla*_KPC-3_ plasmids carried an extra aminoglycoside gene [*aph(3')-Ia*] not present in the 17,266 plasmids (Fig. S4). Like *bla*_KPC-2_, *bla*_KPC-3_ genes were carried on a Tn*4401a* transposon (variant 2), all borne on an IncX3 plasmid.

**Fig 3:**
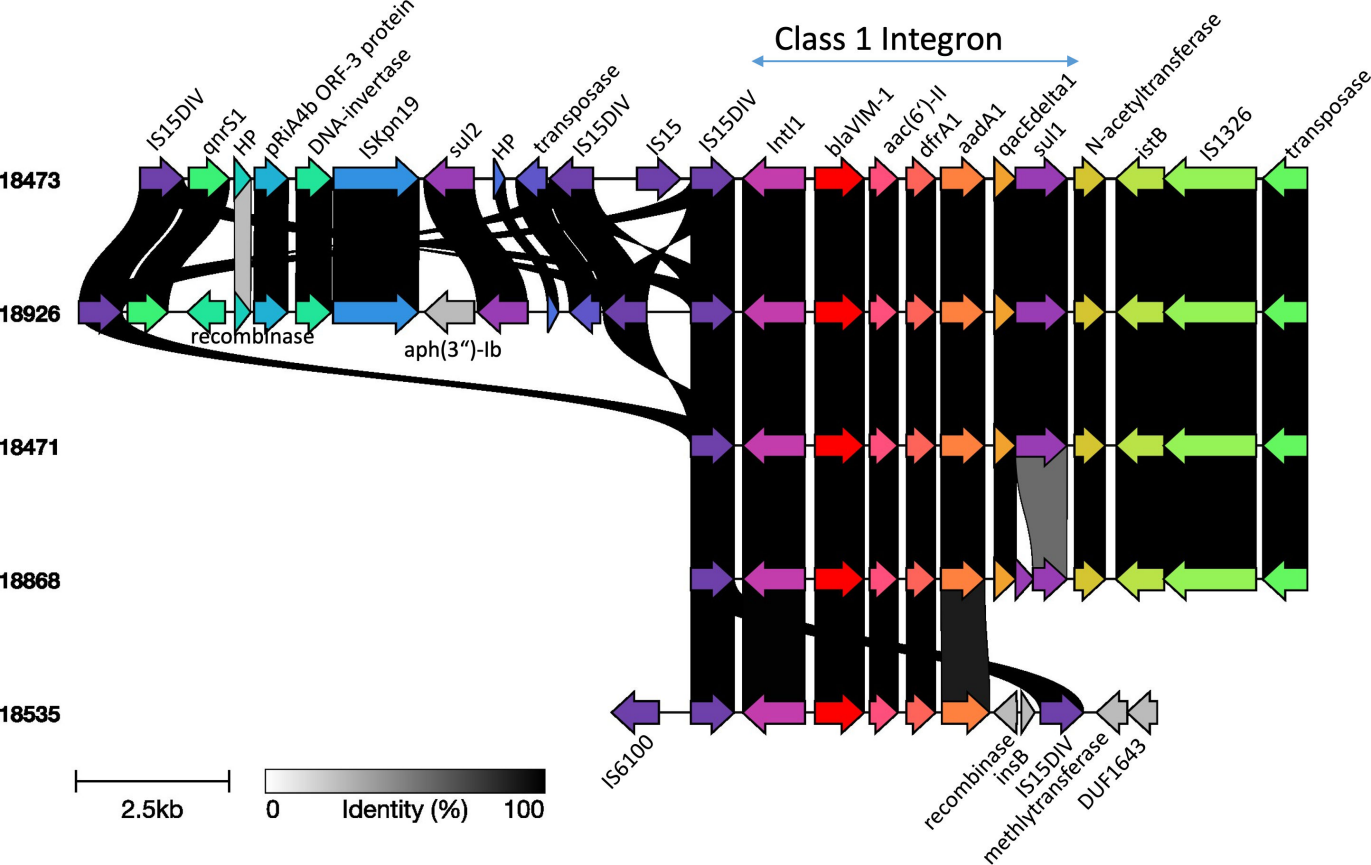
Comparative genomic analysis of selected regions of *bla*_VIM-1_-carrying plasmids belonging to the larger *bla*_VIM-1_ plasmid community. Numbers indicate the isolates’ code number. Red arrows represent the *bla*_VIM-1_ gene. The blue line above the figure defines the class 1 integron region. HP, hypothetical protein.

The *bla*_VIM-1_-carrying plasmids exhibit greater diversity than the *bla*_KPC_-carrying plasmids, as evidenced by the varied genetic contexts of the *bla*_VIM-1_ gene and the broader antimicrobial resistance gene repertoire (Fig. 1 and Plasmid Microreact link). Additionally, the structural evolutionary relatedness between the circularized *bla*_VIM-1_ plasmids (Fig. S2B and C) further underscores their diversity. The larger *bla*_VIM-1_ plasmid community is composed of IncC plasmids, while the smaller community consists of plasmids characterized by a fusion of the IncFIB(pNDM-Mar) and IncHI1B(pNDM-MAR) plasmid types (Fig. 1 and Microreact link). Despite this diversity, three out of the four *bla*_VIM-1_-carrying IncC plasmids in one sub-community (Fig. S5) were identical and were derived from strains carrying double-carbapenemase genes belonging to sequence types 39 and 258 (plasmid size range: 155–165 kbp; copy number: 1.76–1.98). This suggests a probable transmission of *bla*_VIM-1_ plasmids within and across Kp lineages. Of note, we observed that *bla*_VIM-1_ genes were always located on class one integrons (Fig. 3; Fig. S6).

## DISCUSSION

The acquisition of multiple carbapenemase genes by “high-risk” clones of Kp is an emerging public health threat. The majority of these isolates belong to a small number of high-risk clones; ST147, ST258, ST39, and ST323. In particular, the clones ST147 and ST258 have been established in our healthcare facility since 2003 and 2006 respectively whereas the clones ST323 and ST39 were observed sporadically until 2019 and currently are expanding and spread in major hospitals across the country (10, 11). Although all these clones had been detected in our hospital for a prolonged period of time (10), none of these contained two different carbapenemase genes before 2018, with the exception of one isolate that belonged to ST340 and carried both *bla*_KPC-2_ and *bla*_VIM-1_.

By comparing the plasmid content of isolates belonging to lineages ST340, ST258, ST147, ST323 carrying originally one carbapenemase gene, VIM-1, KPC-2 or KPC-3, VIM-1 or KPC-2, and VIM-1 respectively, with those of the same lineage carrying two carbapenemase genes, our analysis demonstrated that the acquisition of a second carbapenemase gene was mediated by possession of a new plasmid while the plasmid carrying the first carbapenemase gene persisted over time in all isolates examined with the exception of one isolate of ST323 lineage. It is possible that the latter lineage first entered our service with a FIB-HI1B plasmid carrying the *bla*_VIM-1_ gene and in a later entry it presented another plasmid arrangement having acquired two new plasmids, one IncC type carrying *bla*_VIM-1_ and a fusion of FIB-FII plasmid carrying *bla*_KPC-2_, an event that probably rendered the ST323 clone more successful and easier to spread. Apparently, events of acquisition and loss of plasmids carrying carbapenemase genes or other antimicrobial resistance determinants operate frequently alongside the gradual accumulation of resistance in the hospital setting. The addition of a second plasmid may increase the resistance spectrum overall depending on the background population. *Bla*_KPC_ plasmids often only carry this one carbapenemase, whereas *bla*_VIM_ plasmids carry a number of different resistance genes. Acquisition of *bla*_KPC_ into a ST147-*bla*_VIM_ background therefore does not alter the resistance profile significantly, whereas acquisition of *bla*_VIM_ into a ST39-*bla*_KPC_ background adds an average of 5 resistance genes.

During the last three decades, the carbapenemase-encoding genes have distributed across a variety of plasmids (25), which through transmission within the same lineage and between lineages have played a pivotal role in the evolution of epidemiology of CR-Kp worldwide. In our setting, the plasmids carrying the carbapenemase-encoding genes were classified into four groups. The first group consisted of a fusion of IncFIB(pQil) and IncFII(K) plasmid types carrying *bla*_KPC-2_ which are identical to the reference plasmids NZ_CP059315.1 and MN657251.1, both from Kp. The latter was implicated in a polyclonal, multi-species outbreak in a German hospital from 2015 to 2017 (26). Given that conserved plasmids from unrelated sources are indistinguishable (NZ_CP059315) or very similar (CP067719), it is unknown whether these plasmids are shared within the local population or whether there are other externally contributing sources. The second group belonged to IncX3 type carrying *bla*_KPC-3_ which is identical to the *bla*_KPC-3_-carrying IncX3 plasmid (NZ_CP083703.1) from an *Escherichia coli* strain recovered from a patient’s urine in Switzerland. This suggests a broad dissemination of this plasmid across multiple bacterial species. The third group belonged to IncC type carrying *bla*_VIM-1_ and the fourth consisted of a fusion of IncFIB(pNDM-Mar) and IncHI1B(pNDM-MAR) plasmid types harboring also *bla*_VIM-1_. Identical plasmids were found not only in the same bacterial lineage but also across lineages. These findings suggest that dissemination of carbapenemase genes in our hospital setting is mainly driven by multiple plasmids across a variety of highly efficient clones. The “hot spot” of these pathogens was the ICU. However, the frequent transfer of patients between the ICU and hospital wards facilitated further their intra-hospital dissemination.

Although none of these isolates belong to any known virulence capsular type and all exhibit very low virulence scores, the mortality rate of the infected patients was 62.5%, among the highest associated with CR-Kp BSIs (27). Apparently, other factors not related to the virulent traits of the organisms should account for the observed high mortality rate. Indeed, the patient population affected by these pathogens had several distinct characteristics. More than half of the infections occurred in elderly and critically ill patients, many of whom had a number of underlying diseases. Yet, the antibiotic therapy was inadequate as only 2 of the 24 patients received empirical treatment with an *in vitro* active agent during the first 48 h of the infection process, and 5 patients did not receive appropriate therapy even after susceptibility results were available. Thus, the delayed initiation of antimicrobial therapy and the limited therapeutic options in the management of infections caused by organisms producing double carbapenemases had a significant impact on the outcome. Of note, the lowest mortality rate (43%) was observed in the group of patients treated with ceftazidime/avibactam plus aztreonam. In a previous study, the use of this combination resulted in lower mortality of patients infected with MBL-producing *Enterobacterales* compared to treatment regimens based on older antibiotics (28). It is also important to note that the newer agents aztreonam/avibactam and cefiderocol, which recently received approval by regulatory agents, had potent *in vitro* activity in the majority of our isolates. These two agents, along with others in the pipeline, are potential therapeutic options in the management of these difficult-to-treat infections (29); their efficacy, however, remains to be seen in clinical practice.

Our findings should not be interpreted without considering several limitations. First, we examined only blood isolates, not other clinical isolates and isolates from colonization to investigate the extent of dissemination of CR-Kp producing double carbapenemase. Second, the isolates included in our analysis were derived from a single center, and they do not represent the epidemiology of CR-Kp in our geographic region. We also acknowledge the retrospective character of the study and the inherent shortcomings that exist in these types of studies.

The present study underscores the importance of conducting WGS surveillance studies to identify hospital-specific CR-Kp lineages, detect the emergence of “high-risk” clones, and unravel the mechanisms driving AMR dissemination within healthcare facilities. These findings could guide infection control committees in developing strategies to contain the spread of multi-drug-resistant pathogens at the local and national levels and may assist physicians to adopt more effective approaches for management of these difficult-to-treat infections.

## MATERIALS AND METHODS

### Setting and patient population

This is a retrospective study conducted between October 2014 and October 2022 in Laiko General Hospital in Athens, Greece. Laiko is a tertiary-care, 500-bed hospital with 50,000 admissions per year, serving a population of 2 million of the Athens Metropolitan area. During the study period, CR-Kp bloodstream infections have been occurring with incidence ranging from 0.2 to 0.35 per 1,000 patient-days. The patients with bacteremia (≥1 positive blood culture) caused by Kp producing more than one carbapenemase were identified from a laboratory surveillance system. Anonymized clinical and epidemiological data were retrieved from hospital records. Pertinent information including date of positive culture, patient age, gender, hospital ward location of the patient at the time of infection, and outcomes were recorded.

### Microbiology and antimicrobial susceptibility testing

Bacterial isolates underwent identification by Microscan autoSCAN-4 System (Beckman Coulter1260, Nyon, Switzerland) and *in vitro* susceptibility testing by Microscan autoSCAN-4 System and broth microdilution method using Sensititre (Trek Diagnostic Systems, West Syssex, UK). *E. coli* ATCC 25922 and NCTC 13846 were used as control strains. Results were interpreted according to the 2023 European Committee of Antimicrobial Susceptibility Testing clinical breakpoints (v.13.0) (30). Isolates exhibiting a meropenem and/or imipenem MIC of >1 µg/L were examined further for carbapenemase production with the RapidCarba NP test (BioMerieux, MarcyL’Etoile, France). All carbapenemase-producing isolates were stored in skimmed milk at −70°C. Kp blood isolates producing more than one carbapenemase were retrieved from deep refrigeration for WGS.

### WGS and bioinformatic analysis

Genomic DNA was extracted from bacterial colonies using Nucleospin Microbial DNA Kit (Macherey-Nagel, Duren, Germany). The DNA extracts were measured using a Qubit 1X dsDNA HS Assay Kit (Invitrogen; Thermo Fisher Scientific, Waltham, MA, USA) with a Qubit (v.3.0) fluorometer (Invitrogen, Thermo Fisher Scientific). DNA libraries were prepared using a Nextera XT DNA Library Prep Kit (Illumina Inc., San Diego, CA, USA). Sequencing was performed on an Illumina MiSeq Sequencing System (Illumina Inc.). *De novo* assembly was performed with SPAdes (v.3.11.1) (31), and sequence reads were assigned to the corresponding species using the Kraken (v.0.10.5-beta) (32) and the minikraken 4 GB reference databases. Multi-locus sequence typing was performed by mlst v2.10 (33). STs, K and O types, and antimicrobial resistance and virulence genes were identified using Kleborate (v.2.3.2) (34) pipeline (35, 36). Plasmid replicons were identified using Abricate (v.1.0.1) (37) and the PlasmidFinder database (38). MGEs were identified using mefinder (v.1.1.2) (39).

To construct a multi-sequence alignment for genomes belonging to STs 39, 147, 258, and 323, corresponding reads were mapped to the reference assemblies NZ_CP009114.1 (GCF_000739495.1, ST39), JACTAR01 (ST147), CP006923.1 (ST258), and NZ_CP024496.1 (GCF_002752905.1, ST323), respectively, using a publicly available Python script (40) that utilizes ska (41, 42). The reference genomes NZ_CP009114.1 and NZ_CP024496.1 were chosen by Bactinspector (v.0.1.3) as the closest reference for the strains belonging to STs 39 and 323, respectively. Following the construction of a multi-sequence alignment for each ST, the removal of regions resembling MGEs (43), and the extraction of SNP sites using snp-sites (v.2.5.1) (44), the resulting alignment was used to reconstruct a maximum-likelihood phylogenetic tree using RAxML (v.8.2.4) (45). The interactive tree of life (iTOL) annotation files were generated using the itol.toolkit (v.1.1.5) (46) in R (v.4.2.2) (47). Trees and metadata were visualized in iTOL (48) and Microreact (49).

To investigate the location of the second carbapenemase gene, a subset of 16 isolates was selected for long-read genome sequencing on the GridION X5 Mk1 sequencing platform. Eight isolates carrying a single carbapenemase gene from a previous study (10) were matched by sequence types with eight isolates carrying double carbapenemase genes from the present study (ST258, *n* = 2 and 2; ST39, *n* = 2 and 2; ST147, *n* = 2 and 1; ST323, *n* = 1 and 1; and ST340, *n* = 1 and 1, respectively; and ST3035, *n* = 1; no isolate with a single carbapenemase gene was available). Plasmids were assembled from long- and short-read sequence data using Hybracter (v.0.9.0), which, in turn, uses Plassembler (v.1.6.2) (50), employing a hybrid assembly approach (51). Plassembler also estimates mash similarities (52) and plasmid copy numbers and reports the best hit to the PLSDB plasmid database (53). Plasmid assemblies were screened for acquired AMR genes using Kleborate (v.2.3.2) (35) and were annotated with Bakta (v.1.9.3) (54). A phylogenetic tree was constructed from mash distances of carbapenemase gene-carrying plasmid assemblies using Mashtree (v.1.4.6) (55). Mobilization genes were detected, and plasmid mobility was predicted using Mobtyper (v.3.1.9) (56). Plasmid community networks were constructed from circularized carbapenemase gene-carrying plasmids using Pling (v.1.1) (24). These networks were visualized using RCy3 (v.2.18.0) (57) in conjunction with Cytoscape (v.3.10.2) (58). TETyper (v.1.1) (59) was used to examine short-read sequence data for isoforms of the Tn*4401* transposon. Integron Finder tool (v.2.0.5) was used to screen plasmid assemblies for integrons and gene cassettes (60). Plasmid comparisons were visualized using Clinker (v.0.0.29) (61) and BRIG (62). The iTOL annotation files were generated using itol.toolkit (46) in R (v.4.2.2) (47). The phylogenetic tree and metadata were visualized statically in iTOL (48) and interactively in Microreact (49), accessible in reference 63.

## Data Availability

Sequence reads and hybrid assemblies can be found in ENA project PRJEB80309 (new isolates) and PRJEB58216 (long-read data added for previously analyzed isolates). Individual accession numbers can be found in Table S1. Metadata and genetic information about strains and plasmids can be accessed using the following Microreact links: ST39: https://microreact.org/project/4icuXcViz6xGWt3LsJ1Tvm-st39daikosproject ST147: https://microreact.org/project/mXgd7KqrduS1kPNkxS8m7h-st147daikosproject ST258: https://microreact.org/project/1zjJFPBzSSDfQnWCwSp9jW-st258daikosproject ST323:https://microreact.org/project/5rXyVmVJWETfqUmdY8muLV-daikos-st323-project Plasmids: https://microreact.org/project/g57ss6tSs6gbYkCWFeAknk-blavimblakpc2andblakpc3plasmids#nf23-overall-view.

## SUPPLEMENTAL MATERIAL

Supplemental figures aac.01462-24-s0001.docx Figures S1 to S6.

Table S1 aac.01462-24-s0002.xlsx Isolate characteristics.

Table S2 aac.01462-24-s0003.docx Plasmid characteristics.

ASM does not own the copyrights to Supplemental Material that may be linked to, or accessed through, an article. The authors have granted ASM a non-exclusive, world-wide license to publish the Supplemental Material files. Please contact the corresponding author directly for reuse.
