# Supplementary material for: Double carbapenemases in Klebsiella pneumoniae blood isolates: dissemination in a single medical center via multiple plasmids and a variety of highly efficient clones
Source: Antimicrob Agents Chemother. 2025 Feb 3;69(3):e01462-24. doi: 10.1128/aac.01462-24 (PMC11881573; doi:10.1128/aac.01462-24)
Supplement: Supplemental figures — Figures S1 to S6. [file aac.01462-24-s0001.docx]

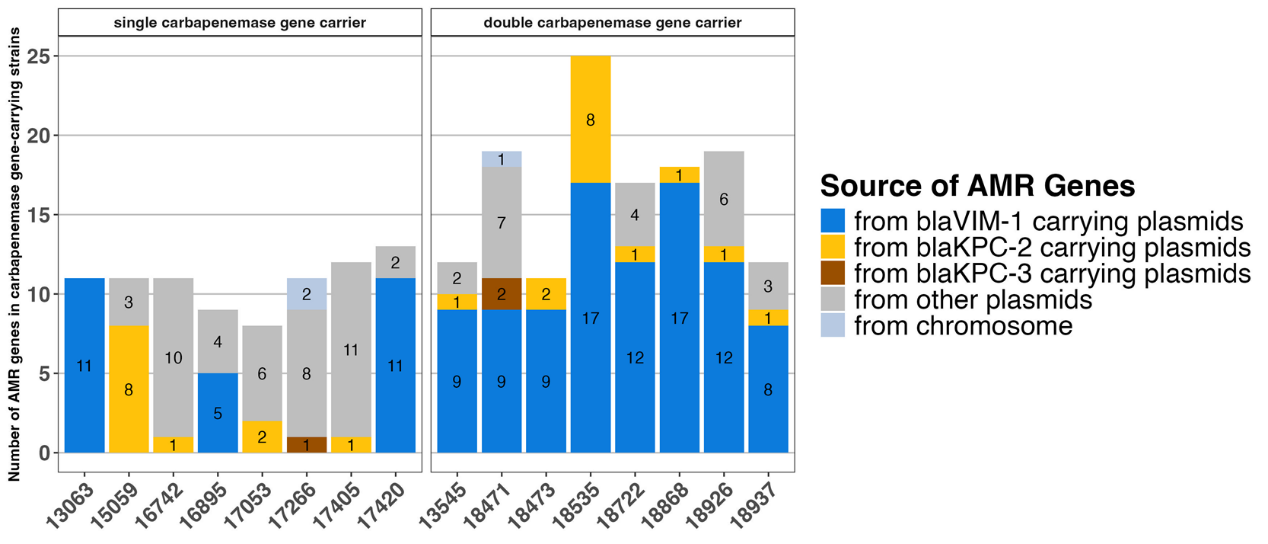


Supplementary Figure 1: Distribution of AMR genes in carbapenemase gene-carrying plasmids, other plasmids, and chromosomes in *K.* *pneumoniae* strains.


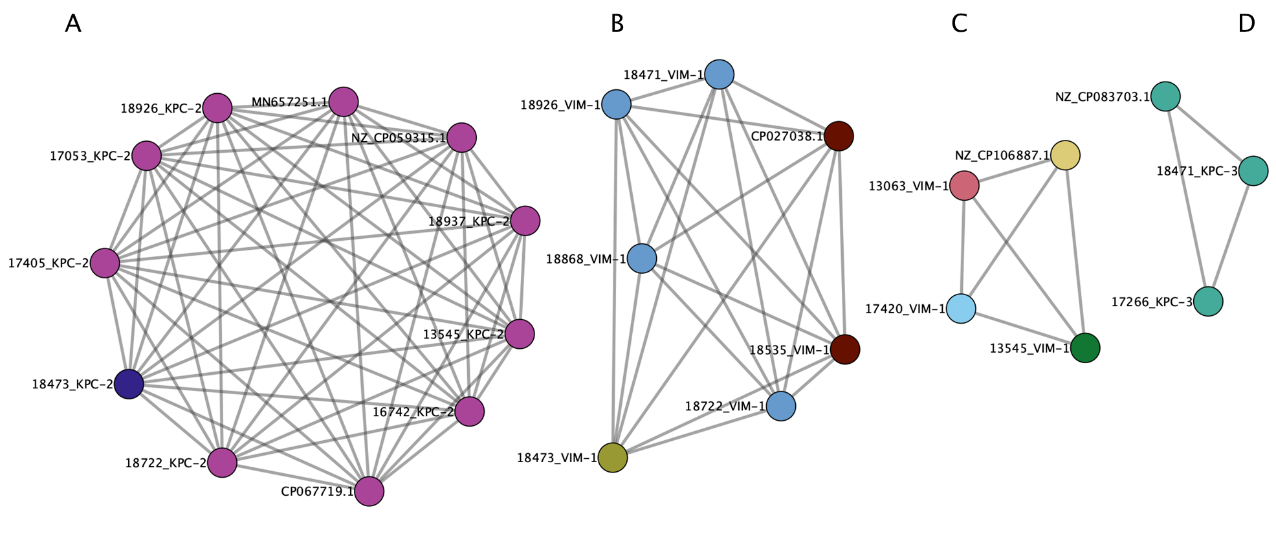


Supplementary Figure 2: Cluster network of carbapenemase gene-carrying plasmids for 16 long-read sequenced *K. pneumoniae* genomes: A, *bla*_KPC-2_ plasmids; B, larger *bla*_VIM-1_ plasmid community; C, smaller *bla*_VIM-1_ community; D, *bla*_KPC-3_ plasmids. Non-circular plasmids are not included in the analysis. Plasmid communities and sub-communities are assigned based on the plasmid containment distance (threshold = 0.3) and double-cut-join-indel distances (threshold = 3). Plasmid sub-communities are indicated by different node colours within each plasmid network.


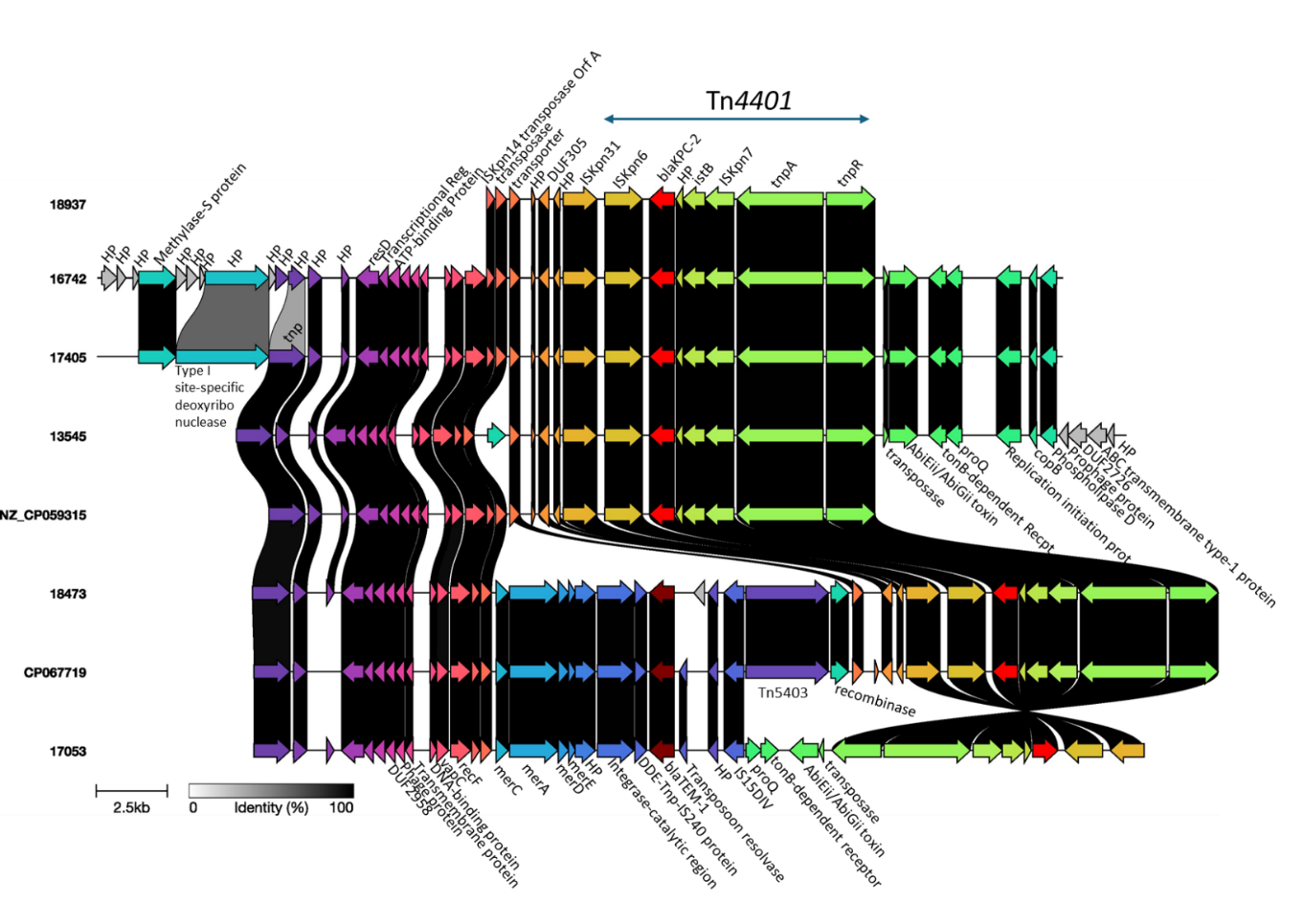


Supplementary Figure 3: Comparative genomic analysis of selected regions of *bla*_KPC-2_-carrying plasmids. Numbers indicate the isolates’ code number. Red arrows represent *bla*_KPC-2_ gene. The blue line above the figure defines the Tn*4401* mobile transposon (composed of *bla*_KPC-2_ gene, transposase gene *tnpA*, resolvase gene *tnpR*, and two insertion sequences IS*Kpn6* and IS*Kpn7*). HP refers to hypothetical protein


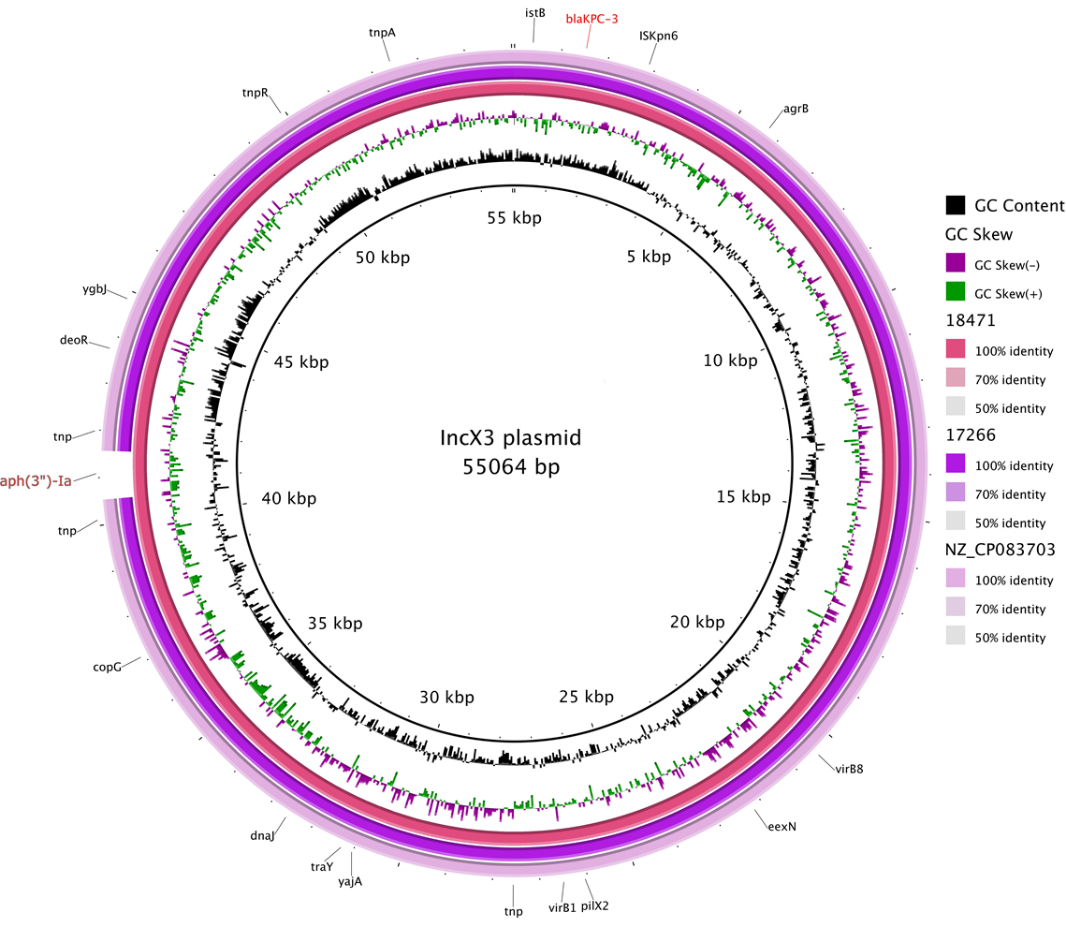


Supplementary Figure 4: Circular maps of *bla*_KPC-3_-bearing plasmids and the reference plasmid NZ_CP083703.1, using blast ring image generator (BRIG). AMR genes (in this case, *bla*_KPC-3_ and *aph(3’)-Ia*) were indicated in red colour, while other genes were indicated in black colour. The innermost red-coloured ring represents the 18471 IncX3 plasmid; the middle ring represents the 17266 IncX3 plasmid, while the outer ring represents the reference plasmid NZ_CP083703.1.


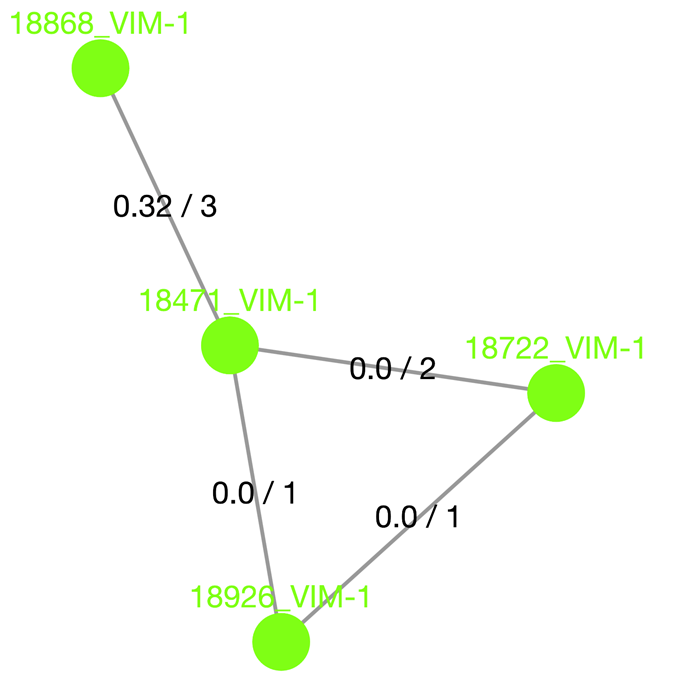


Supplementary Figure 5: blaVIM-1 sub-community comprising more than one identical plasmid. As per Supplementary Figure 2B, only the 18471_VIM-1 plasmid is related to 18868_VIM-1 plasmid (the double-cut-join-indel distance between them is less than 4 structural evolutionary events – the threshold for plasmid sub-community assignment). The other two (18722 and 18926) linked in a triangular network with 18471 plasmid would have formed a separate sub-community from 18868 plasmid had the 18471 plasmid been excluded.


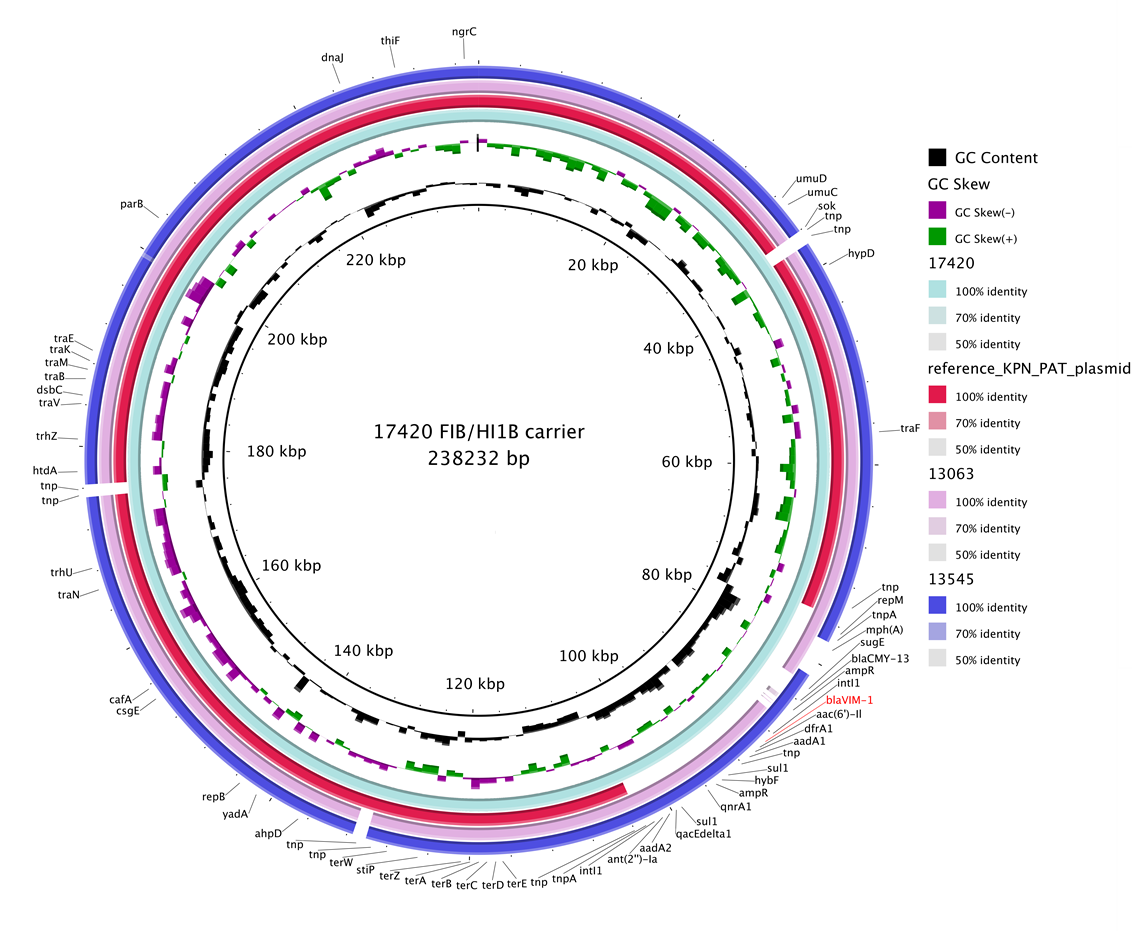


Supplementary Figure 6: Circular maps of *bla*_VIM-1_-bearing plasmids and the reference plasmid NZ_CP106887.1 belonging to the smaller *bla*_VIM-1_ plasmid community, using blast ring image generator (BRIG). Carbapenemase genes (in this case *bla*_VIM-1_) were indicated in red colour. The innermost light-blue ring represents the 17420 IncFIB_IncHI1B plasmid; the next red ring represents the reference plasmid NZ_CP106887.1; the two outermost rings represents the 13063 and 13545 IncFIB_IncHI1B plasmids, respectively
