# Supplementary material for: Double carbapenemases in Klebsiella pneumoniae blood isolates: dissemination in a single medical center via multiple plasmids and a variety of highly efficient clones
Source: Antimicrob Agents Chemother. 2025 Feb 3;69(3):e01462-24. doi: 10.1128/aac.01462-24 (PMC11881573; doi:10.1128/aac.01462-24)
Supplement: Table S2 — Plasmid characteristics. [file aac.01462-24-s0003.docx]

| **Supplementary Table 2.** Characteristics of twenty-four plasmids detected in eight isolates carrying a single carbapenemase gene and eight isolates, matched by sequence type, carrying two carbapenemase genes. | | | | | | | | | | |
| --- | --- | --- | --- | --- | --- | --- | --- | --- | --- | --- |
| Isolate No. | Sequence type | Carbapenemase gene | AMR^a^  genes, n | Plasmid copy number | Size (bp) | Circularity | Inc type (s) | MOB^b^ type(s) | Predicted mobility | Plasmid community |
| 13063 | 340 | *bla*_VIM-1_ | 11 | 1.37 | 401,363 | circular | FIB, HI1B | F,H | conjugative | VIM-1 singletons |
| 13545 | 340 | *bla*_KPC-2_ | 1 | 2.04 | 102,102 | circular | FIB, FII | F | conjugative | KPC-2 subcommunity1 |
|  |  | *bla*_VIM-1_ | 9 | 1.6 | 229,094 | circular | FIB, HI1B | H | conjugative | VIM-1 singletons |
| 15059 | 258 | *bla*_KPC-2_ | 8 | 1.29 | 263,627 | Not circular | FIB, FII | F | conjugative | Unassigned |
| 18535 | 258 | *bla*_KPC-2_ | 8 | 2.73 | 267,659 | Not circular | FIB, FII | F | conjugative | Unassigned |
|  |  | *bla*_VIM-1_ | 17 | 2.29 | 160,869 | circular | C | H | conjugative | VIM-1 singletons |
| 17266 | 258 | *bla*_KPC-3_ | 1 | 0.62 | 53,292 | circular | X3 | P | conjugative | KPC-3 subcommunity |
| 18471 | 258 | *bla*_VIM-1_ | 9 | 1.98 | 155,574 | circular | C | H | conjugative | VIM-1 subcommunity |
|  |  | *bla*_KPC-3_ | 2 | 2.18 | 55,064 | circular | X3 | P | conjugative | KPC-3 subcommunity |
| 16742 | 39 | *bla*_KPC-2_ | 1 | 0.93 | 100,948 | circular | FIB, FII | F | conjugative | KPC-2 subcommunity1 |
| 17405 | 39 | *bla*_KPC-2_ | 1 | 0.86 | 100,960 | circular | FIB, FII | F | conjugative | KPC-2 subcommunity1 |
| 18722 | 39 | *bla*_VIM-1_ | 12 | 1.76 | 166,888 | circular | C | H | conjugative | VIM-1 subcommunity |
|  |  | *bla*_KPC-2_ | 1 | 2.12 | 100,959 | circular | FIB, FII | F | conjugative | KPC-2 subcommunity1 |
| 18926 | 39 | *bla*_VIM-1_ | 12 | 1.82 | 165,575 | circular | C | H | conjugative | VIM-1 subcommunity |
|  |  | *bla*_KPC-2_ | 1 | 1.89 | 100,962 | circular | FIB, FII | F | conjugative | KPC-2 subcommunity1 |
| 16895 | 147 | *bla*_VIM-1_ | 5 | 1.06 | 10,014 | Not circular | - | - | non-mobilizable | Unassigned |
| 17053 | 147 | *bla*_KPC-2_ | 2 | 0.97 | 124,614 | circular | FIB, FII | F | conjugative | KPC-2 subcommunity1 |
| 18937 | 147 | *bla*_KPC-2_ | 1 | 2.18 | 86,551 | circular | FIB, FII | F | conjugative | KPC-2 subcommunity1 |
|  |  | *bla*_VIM-1_ | 8 | 1.61 | 13,756 | Not circular | - | - | non-mobilizable | Unassigned |
| 17420 | 323 | *bla*_VIM-1_ | 11 | 0,88 | 238,232 | circular | FIB, HI1B | H | conjugative | VIM-1 singletons |
| 18868 | 323 | *bla*_KPC-2_ | 1 | 2.59 | 212,430 | Not circular | FIB, FII | F | conjugative | Unassigned |
|  |  | *bla*_VIM-1_ | 17 | 1.64 | 132,295 | circular | C | - | mobilizable | VIM-1 subcommunity |
| 18473 | 3035 | *bla*_VIM-1_ | 9 | 1.5 | 184,232 | circular | C | H | conjugative | VIM-1 singletons |
|  |  | *bla*_KPC-2_ | 2 | 2.3 | 126,011 | circular | FIB | F | conjugative | KPC-2 subcommunity 2 |

a, AMR = antimicrobial resistance; b, MOB = mobilization
